# The Architectonic Experience of Body and Space in Augmented Interiors

**DOI:** 10.3389/fpsyg.2018.00375

**Published:** 2018-04-10

**Authors:** Isabella Pasqualini, Maria Laura Blefari, Tej Tadi, Andrea Serino, Olaf Blanke

**Affiliations:** ^1^Laboratory of Cognitive Neuroscience, Brain Mind Institute, École Polytechnique Fédérale de Lausanne, Geneva, Switzerland; ^2^Atelier de la Conception de l’Espace, Institute of Architecture and the City, École Polytechnique Fédérale de Lausanne, Lausanne, Switzerland; ^3^Defitech Chair in Brain-Machine Interface, Institute of Bioengineering, École Polytechnique Fédérale de Lausanne, Geneva, Switzerland; ^4^Center for Neuroprosthetics, École Polytechnique Fédérale de Lausanne, Geneva, Switzerland; ^5^Department of Psychology, Alma Mater Studiorum – University of Bologna, Bologna, Italy; ^6^Department of Neurology, Geneva University Hospital, Geneva, Switzerland

**Keywords:** self-consciousness, body ownership, embodiment, architecture, augmented space

## Abstract

The environment shapes our experience of space in constant interaction with the body. Architectonic interiors amplify the perception of space through the bodily senses; an effect also known as embodiment. The interaction of the bodily senses with the space surrounding the body can be tested experimentally through the manipulation of multisensory stimulation and measured via a range of behaviors related to bodily self-consciousness. Many studies have used Virtual Reality to show that visuotactile conflicts mediated via a virtual body or avatar can disrupt the unified subjective experience of the body and self. In the full-body illusion paradigm, participants feel as if the avatar was their body (ownership, self-identification) and they shift their center of awareness toward the position of the avatar (self-location). However, the influence of non-bodily spatial cues around the body on embodiment remains unclear, and data about the impact of architectonic space on human perception and self-conscious states are sparse. We placed participants into a Virtual Reality arena, where large and narrow virtual interiors were displayed with and without an avatar. We then applied synchronous or asynchronous visuotactile strokes to the back of the participants and avatar, or, to the front wall of the void interiors. During conditions of illusory self-identification with the avatar, participants reported sensations of containment, drift, and touch with the architectonic environment. The absence of the avatar suppressed such feelings, yet, in the large space, we found an effect of continuity between the physical and the virtual interior depending on the full-body illusion. We discuss subjective feelings evoked by architecture and compare the full-body illusion in augmented interiors to architectonic embodiment. A relevant outcome of this study is the potential to dissociate the egocentric, first-person view from the physical point of view through augmented architectonic space.

## Introduction

Architecture shelters and constrains the daily experience of our body in space. It is therefore not surprising that the adaptation of architectonic forms to the bodily senses evolved as a central architectonic theme over centuries. Several scholars proposed that beyond the architectonic composition with modules, that is, walls, columns, vaults and so on, visual cues were introduced to augment the experience of continuity in physical space (see **Box 1**). For instance, the striving toward the effect of spatial unity can be seen in the application of color, texture or sculpture to architectonic interiors, with the aim to elicit a more elaborated degree of complexity in the experience of space. The question then, how spatial sensations, such as feelings of depth, continuity, presence, vertigo, containment, safety or familiarity, can be associated with meaning as well as emotion, has occupied vast amounts of literature. Embodiment theories in architecture have widely claimed that a figurative or abstract representation of the human body enhances sensations related to the architectonic environment (Alberti, 1450/1988; Marcus Vitruvius Pollio, 2008) (see **Box 2**).

**FIGURE 1:**
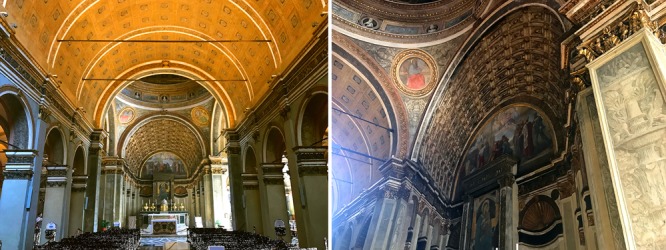
Santa Maria presso San Satiro, Milan, was completed by Donato Bramante in 1482. The first view shows the built nave in the foreground (picture on the left). When approaching, the chancel’s interior disappears giving view to a relief painting inside the arch segment behind the altar.

**Box 1.** Linear perspective and the ideal of continuity. The two perspective demonstrations performed in the 15th century by the architect Filippo Brunelleschi unveiled a unified and embodied viewpoint in space linked to its pictorial representation, rendering the perfect illusion of space at “one, and one point only” (Manetti, 1480/1970; Lindberg, 1976). Linear perspective was since then in use to augment architectonic space with the sensation of continuity (Alberti, 1435/2011). The architect and painter Leon Battista Alberti deemed it less apt as a tool for the architectonic composition itself, considering its particular relation to one specific point of view in space (Alberti, 1450/1988). The gradual evolution of perspective and its application to the classical module has been seemingly motivated by the ideal of a continuous or infinite space, as the central concern of Renaissance art (Burckhardt, 1868; Argan, 1946; Wittkower, 1953). Two famous examples by the architect Donato Bramante illustrate the double purpose of linear perspective to augment and shape the classic module. A first example is the pictorial augmentation of the chancel at Santa Maria presso San Satiro (**Figure 1**); a second one, the Belvedere courtyards at the Vatican, where only from one window of the “Stanze” (pope’s apartment) the top view is complemented geometrically by the design of the gardens, intended to evoke a feeling of association between the worldly and the divine (Vasari, 1550/1986).

**FIGURE 2:**
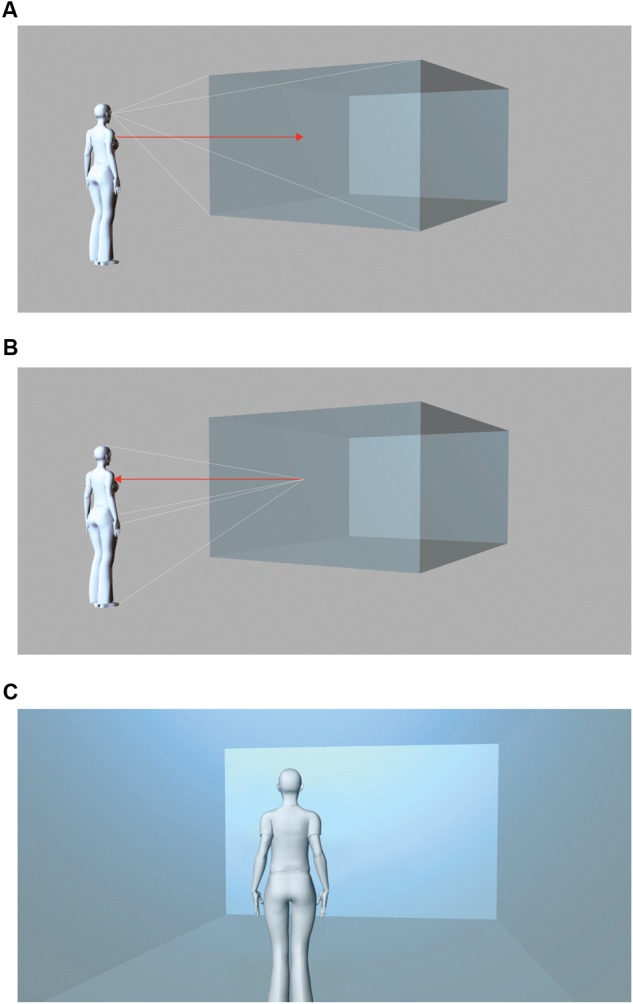
**(A)** ‘Sympathetic transposition’ of feelings toward form as described by Hermann Lotze and Robert Vischer. **(B)** ‘Kinesthetic’ reverberation of architectonic form inside the human body after Heinrich Wölfflin. **(C)** An ‘objectified’ perspective of the observer within space generates a ‘sense of Space,’ as described by August Schmarsow.

**Box 2.** Architectonic embodiment and ‘Einfühlung’. The theories of architectonic embodiment propose since millennia that through the specific use of proportion the human sensation can be embedded into the environment. The most ancient theory of embodiment known by architects has been written by Vitruvius (Vitruvius, 1st century BC). It is based on Democritic philosophy and articulates a formal appropriation of the architectonic module through the senses. Importantly, in the Quattrocento Alberti evolved this idea further, based on the work by Filippo Brunelleschi, who studied the geometric proportions of the architectonic ensemble and its spatial effects on the human body through linear perspective (Alberti, 1450/1988, see **Box 1**). Proposing an everlasting ‘Style’, the architect Gottfried Semper applied the anthropological approach to the theory of embodiment by comparing the architectonic interior module to a woven cloth or a molded receptacle (Semper, 1860/2004). This idea was at the roots of a debate about *Einfühlung* at the turn of the 19th century, that is, the ‘sympathetic transposition’ of feelings to form as proposed by Hermann Lotze and Robert Vischer (**Figure 2A**) (Vischer, 1872/1994; Lotze, 1884). Heinrich Wölfflin suggested an observer *in front* of the architectonic module resonating through a ‘kinesthetic’ transposition in the bodily members (**Figure 2B**) (Wölfflin, 1886/1994). August Schmarsow imagined an observer completely immersed *within* space (**Figure 2C**) (Schmarsow, 1893/1994). The central question of these theories was related to the transmission of cultural ideals, such as continuity (see **Box 1**). In a famous quote, Wölfflin claimed that to see an asymmetric building evokes the same feelings “as if a limb was missing” (Wölfflin, 1886/1994). Two decades later, *Einfühlung* was translated as empathy (Lipps, 1903), meaning the ability of putting oneself into another person’s position.

Modern embodiment theory compared the stylistic influence of the architectonic envelope, the *Wand*, on the human body to a cloth or *Ge-wand* (Semper, 1860/2004). In the attempt to unify the theories of style through the association of perception and form, art historians have often introduced notions of empirical science. For instance, August Schmarsow mentioned a sensation of space or *Raumgefühl* (**Figure 2C**), through the immanent feeling of the presence of the body in space (Schmarsow, 1893/1994). For Heinrich Wölfflin, the muscular repercussions within the bodily limbs provoked an architectonic mood, a so-called ‘kinesthetic’ response of the body to the structural elements seen in the environment, or, in his words, *Einfühlung*, a ‘feeling into’ the form based on a ‘kinesthetic’ projection that exceeded the purely retinal effects of vision (Wölfflin, 1886/1994) (**Figures 2A,B**). These ‘kinesthetic’ reverberations that were mediating between the human body and aspects of verticality, orientation, and symmetry in the architectonic composition, have been related to a minimal form of embodiment, that is, sensorimotor mechanisms of visual perception, namely eye- or head-movements (Pasqualini and Blanke, 2014).

On the other hand, phenomenological theory in architecture relates spatial effects of embodiment to the ‘presence’ of a building (Norberg-Schulz, 1980); to a multisensory image (Neutra, 1954; Pallasmaa, 1996); or, to architectonic ‘atmospheres’ (Böhme, 2006; Zumthor, 2006). Other lines of theory based on visuospatial phenomena in cognitive science and Gestalt theory, link embodiment to visuomotor affordance (Gibson, 1979/1986; Oztop and Arbib, 2002), or, to the interplay of the body with spatial configurations (Arnheim, 1977). Affordance and its multisensory dimension based on bi-modal visuomotor integration have also been related to ‘mirror neurons’ and more in general to ‘mirror’-like or resonance mechanisms in the brain (di Pellegrino et al., 1992; Rizzolatti and Craighero, 2004). By ‘mirror’-like mechanisms, we refer to multimodal areas in the brain that respond not only when performing a movement, but also when observing or hearing the same movement (Thill et al., 2013). Such ‘mirror’-like or resonance mechanisms generalize to other sensory modalities, such as somatosensation (Keysers and Gazzola, 2009; Keysers et al., 2010), or emotion, including, for instance, pain processing and disgust (Jackson et al., 2006; Lamm et al., 2011). In this respect, it has been proposed that the emotional reaction to artworks is elicited by a ‘mirror’-like response of the brain to the representation of visuomotor processes (Freedberg and Gallese, 2007).

When, instead of the perception of artworks, we focus on the perception of architectonic spaces, the embodiment effect extends to the subjective sense to own a body at a precise location, based on multisensory representations. In seminal texts of psychology and philosophy, the continuous and unified self-conscious experience of the body in space is considered a prerequisite to perceive the external world (James, 1890/1950; Merleau-Ponty, 1945/2002). Neuroscientists distinguish between multisensory representations of the space immediately surrounding the body, where we can physically interact with external objects – termed peripersonal space, from the space further away from the body – termed extrapersonal space, prima facie accessible only through distant senses like vision and audition (Grüsser and Landis, 1991; Rizzolatti et al., 1997; Graziano and Cooke, 2006; Ladavas and Serino, 2008). Under normal circumstances, the experience of oneself is therefore bound to that of one’s body within peripersonal space (i.e., Blanke et al., 2015). Authors from several disciplines converge on the fact that the experience of one self in space depends on the integration of multisensory and sensorimotor inputs from the body and the peripersonal space, that is, visual, tactile, vestibular, proprioceptive, auditory, and interoceptive (Blanke et al., 2004; Gallagher, 2005; Legrand, 2006; Gallese and Sinigaglia, 2010; Tsakiris, 2010; Serino et al., 2013, Pfeiffer et al., 2014b).

The experience of one self in space through the body has been defined as bodily self-consciousness (Blanke and Metzinger, 2009). It has been further decomposed in the experience of feeling one’s physical body as one’s own (body ownership or self-identification), while being at a specific location in space (self-location), and of facing the external world from a unified, embodied perspective (first-person perspective) (Blanke et al., 2000, 2002, 2015; Blanke, 2004, 2012). Interestingly, bodily self-consciousness can be dissociated from the physical body through the application of visuotactile conflicts between the physical own body (or body parts) and an artificial replacement of it, such as a virtual body (or body parts) (Botvinick and Cohen, 1998; Ehrsson, 2007; Lenggenhager et al., 2007, 2009; Tsakiris et al., 2007; Petkova and Ehrsson, 2008; Aspell et al., 2009; Slater et al., 2010; Tsakiris, 2010). For instance, in the so-called full-body illusion (for details see Lenggenhager et al., 2007), participants are filmed from behind, and the filmed scene is projected on a Head-Mounted-Display. While participants are stroked on their back with a stick, they can watch on the Head-Mounted-Display their virtual body or avatar being stroked at a distance of two meters, either in synchrony (real-time) or in asynchrony (delayed) with the felt tactile stimulation. In the synchronous, as opposed to the asynchronous condition, participants report the feeling of self-identifying with the avatar. When asked to indicate the position of their body within the environment, participants exhibit a drift in self-location toward the avatar. When a human-sized box is shown instead of the avatar these illusory effects disappear.

One intriguing question is, how multisensory and sensorimotor mechanisms underlying feelings of body ownership and bodily self-consciousness interact with the experience of the surrounding space. In other words, how does architectonic space influence bodily self-consciousness? And does the environment impact our feelings and behaviors depending on how the body is embedded into the environment as claimed by architects? Previous studies highlight a relationship between external space and bodily self-consciousness during multisensory bodily illusions. For instance, the strength of illusory effects has been shown to depend on proximity (Lloyd, 2007), and visuospatial congruency between one’s own and the artificial body (Graziano et al., 2000; Pavani et al., 2000; Costantini and Haggard, 2007; Blanke et al., 2015). On the other hand, it has been shown that the perception of dimensions, inclination, size, and weight of the elements in an environment, such as the slant of hills, depends not so much on an objective estimation of their physical characteristics in terms of their interaction potential or affordance. Instead, it relies mainly on a subjective bias induced by the perceived size or weight of the own body (Proffitt, 2006; Witt and Proffitt, 2007; Linkenauger et al., 2011). This view has been linked to ownership feelings of the body, providing the evidence that the perceived size of the environment is affected by the size of the body, with which the participants identify. Van der Hoort et al. (2011) induced illusory ownership sensations using visuotactile stimulation of an avatar that appeared either as too small or as too big and found that participants judge both, distance and size of the surrounding objects depending on the size of the avatar they identify with. Related embodiment effects of an avatar were also linked to pain processing (Romano et al., 2014).

Up to today only a few studies explicitly addressed the relationship between multisensory aspects of bodily self-consciousness and the external environment. To this extent, another study employs a video-based setup as used by Lenggenhager et al. (2007), while manipulating the size of the external environment (Pasqualini et al., 2013). In this experiment, the full-body illusion was induced with an avatar standing in two different interiors. The interiors were simulated in a robotic space with a flexible wall to generate either a large or narrow space. After each experimental condition participants performed size estimations of visual stimuli that were placed in the interiors. The results of this study prove that the full-body illusion stimulates embodiment in virtual interiors and affects depth perception. This effect is modulated by the dimensions of the interiors. From this and previous experiments though, it remains unclear how the presence of an avatar alters the way in which we perceive the environment, and, in turn, how the interior by itself modifies the experience of one’s body.

**FIGURE 3:**
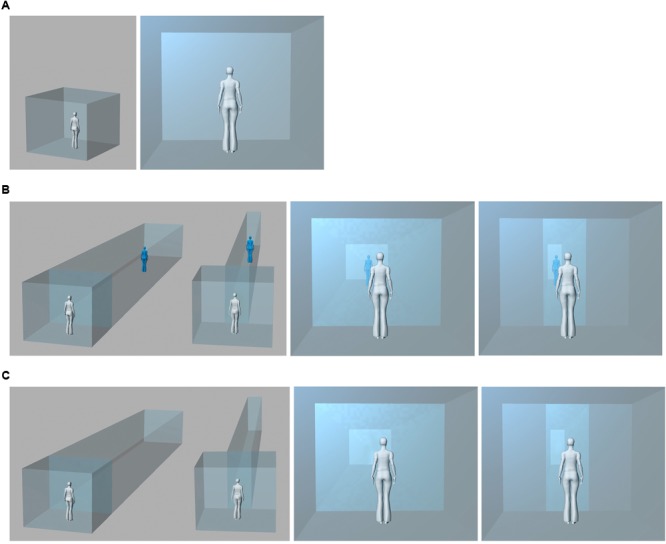
The experimental setup. **(A)** The physical interior of the Virtual Reality arena: A Participant standing inside the Virtual Reality arena without virtual extension; her view in first person from behind the physical body oriented toward the rear-projection screen. **(B)** The physical interior and its virtual augmentation in Experiment 1: The participant stands inside the Virtual Reality arena with a large and narrow virtual interior extension; first person view of the augmented interior with an avatar (blue) and the physical body (white) from behind. **(C)** The physical interior and its virtual augmentation in Experiment 2: The participant stands inside the Virtual Reality arena with a large and narrow virtual interior; first person view of the augmented interior without the avatar and the physical body (white) from behind.

The aim of the present study, is to investigate the effects of the interior room size on the bodily self in space. In Experiment 1, we manipulated the width of the virtual room as in the previous study (Pasqualini et al., 2013), along with the visuotactile congruency between the tactile stimulation of the participant’s physical own body (back) and the visual stimulation of the virtual body (back) or the void interior (front wall). Participants were standing in a Virtual Reality arena (**Figure 3**). They were looking at the back of an avatar that was presented either in a large virtual interior with the sidewalls in the extrapersonal space of the avatar, or, in a narrow interior with the sidewalls in the avatar’s peripersonal space. Participants were exposed to visuotactile stimulation in a two-by-two factorial design, with a combination of multisensory inputs (i.e., synchronous or asynchronous stimulation) and architectonic space (i.e., large or narrow interior space). In Experiment 2, to test whether the induced changes in bodily self-consciousness depended on self-identification with a body in the virtual environment, we presented no avatar and showed the visual stroking (associated to the tactile stroking of the back) on the front wall of the virtual interior. Through questionnaires, we measured how the association of multisensory and architectonic cues impacts bodily self-consciousness and how the changes of bodily self-consciousness influence the subjective experience of the architectonic interiors.

We hypothesized an interaction between stimulation pattern and interior dimensions, suggesting the presence of fundamentally distinct mechanisms of perception for the large and the narrow interior. Differing from previous studies that had tested the full-body illusion without perspective cues (see Lenggenhager et al., 2007), or, which introduced perspective cues but only in a setup with a body (see Pasqualini et al., 2013), here we exposed participants to equivalent perspective cues, with and without virtual body. Inside the narrow interior, in combination with the virtual body, we expected a stronger interference of multisensory perception with the environment, enhancing illusions of touch and drift but also enabling depth perception through the full-body illusion (see also Pasqualini et al., 2013 on this behalf). For the large interior, we assumed a more visual response resulting in the incorporation of the global interior volume and a weaker interference with the virtual body. This fundamental difference of depth feelings was supposed to be revealed by the second study, where we expected low ratings for depth and immersion for the narrow interior, and higher ones for the large space and synchronous stimulation with the front wall.

## Materials and Methods

### Experiment 1

#### Participants

A total of 19 healthy, right-handed participants were selected for Experiment 1 (mean age 27.2 ± 8.8 years; 13 females). The sample size for Experiment 1 was estimated from power analysis of prior studies (Lenggenhager et al., 2007, 2009), which required 18 participants for a power of 0.8. One participant was excluded due to incomplete questionnaires. Participants had neither previous experience with the task, nor had they performed similar experimental paradigms. All participants had normal or corrected to normal vision and no history of neurological or psychiatric conditions. Participants gave written and informed consent before the experimental study and were paid 20 Swiss Francs per hour. The study protocol was approved by the local ethics research committee – La Commission d’Ethique de la Recherche Clinique de la Faculté de Biologie et de Médecine at the University of Lausanne, Switzerland and was performed in accordance with the ethical standards in the Declaration of Helsinki.

#### Materials

Participants were placed at the center of the Virtual Reality arena (3 m × 3 m × 2.50 m) (**Figure 3A**) equipped with an active tracker system (ReActor 2; Ascension Technology, Burlington, VT, United States, capture rate of 30 Hz). They were facing a rear-projection screen (projection area: 3.2 m wide × 2.35 m high) on which an architectonic interior was rendered, either large (same as the physical space) or narrow (walls in the reaching space of the avatar). The physical sidewalls of the Virtual Reality arena were covered with dark cloth to ensure the continuity between virtual and physical interiors. The sidewalls were visibly confining the interior, matching it to the perspective view during the experimental conditions. We presented a life-sized back-facing avatar within the virtual extensions (**Figure 3B**). To administer stroking, we used a stick on which an optical marker was mounted to track the stroking movement with infrared cameras. We manipulated bodily self-consciousness by stroking the back of the participant and the avatar in synchronous or asynchronous visuotactile mode (as in Lenggenhager et al., 2007). For synchronous stroking the captured motion data of the marker was projected onto the screen in real time; asynchrony was produced through a stroking delay.

To quantify the strength of the illusion, we used an eight items bodily self-consciousness questionnaire adapted from Lenggenhager et al. (2007). It measured *tactile sensation* (Question A1: “I could locate the touch of the stick in the location where I saw the virtual body being touched”; Question A2: “The touch I felt was caused by the stick touching the virtual body”); *self-identification* with the virtual body (Question A3: “The virtual body was my body”); as well as *self-location* (Question A4: “My physical body was drifting toward the front (toward the virtual body)” (on illusory drift and self-location see Lenggenhager et al., 2009; Serino et al., 2013; Noel et al., 2015; Salomon et al., 2017). Moreover it included four control questions (Question A5: “I might have more than one body”; A6: “The touch I felt came from somewhere between my own body and the virtual body”; A7: “The virtual body was drifting backward (toward my own body)”; and A8: “I was in two places at the same time”) (**Table 1A**). Set-up and aims of this study are different from the original paper on the full-body illusion (Lenggenhager et al., 2007). Here, we studied the effects of the architectonic space modulating illusory changes in bodily self-consciousness, and, self-location in particular. According to our main hypothesis, the presence of an architectonic context and its features alters the classic changes in bodily self-consciousness induced by the full-body illusion. Specifically, given the nature of the spatial manipulation implemented, we expected different changes in self-location. For this reason, in the present study, questions referred to self-location (such as questionA4: “My physical body was drifting toward the front - toward the virtual body”) were not considered control questions, but actually questions of interest.

**Table 1:** Questionnaires. Bold letters: means of responses *p* < 0.05.

(A) Self-consciousness questionnaire in Experiment 1, as in Lenggenhager et al. (2007)	
During the Illusion I felt that...	
**A1**	**..I could locate the touch of the stick in the location where i saw the virtual body being touched**
**A2**	**..the touch i felt was caused by the stick touching the virtual body**
**A3**	**..the virtual body was my body**
**A4**	**..my physical body was drifting toward the front (toward the virtual body)**
A5	..i might have more than one body
A6	..the touch i felt came from somewhere between my own body and the virtual body
**A7**	**..the virtual body was drifting backward (towards my own body)**
A8	..i was in two places at the same time
**(B)** Architecture questionnaire in Experiment 1	
During the Illusion i felt that...	
**B1**	**..i was standing inside a corridor**
B2	..i was standing within the same interior space all the time
B3	..i was moving along the interior space
B4	..certain areas within the interior space were located further away from me
**B5**	**..the sidewalls were located closer to me than other parts of the virtual Interior space**
**B6**	**..some elements enclosing the virtual interior space were touching my body**
B7	.. the ceiling and the ground were located very close to me
**B8**	**..every element of the interior space was equally far from me**
B9	..i was standing in an open space
B10	..i was standing outside the virtual interior space
B11	..i was standing in several places at the same time
B12	..i was perceiving the virtual interior space and myself from the outside
**(C)** Mixed questionnaire for the interior view without an avatar in Experiment 2	
During the Illusion I felt that...	
**A1**	**..I could locate the touch of the stick in the location where I saw the virtual wall being touched**
A2	..the touch I felt was caused by the stick touching the virtual wall
A3	..the virtual interior space was part of me
A4	.. my physical body was drifting toward the virtual front wall
**A6**	**..the touch I felt came from somewhere between my own body and the virtual front wall**
A8	..I was in two places at the same time
B6	..some elements enclosing the virtual interior space were touching my body
B9	..I was standing in an open space

Also, we developed a novel twelve items architecture questionnaire based on a previous study on architectonic interiors (Pasqualini et al., 2013), to relate embodiment to visuotactile synchrony through the avatar, or, specific elements of the interiors. The experience of the interior space was assessed through control questions of *place* and *presence* (Question B1: “I was standing inside a corridor”; Question B2: “I was standing within the same interior space all the time”; Question B9: “I was standing in an open space”; Question B10: “I was standing outside the virtual interior space”; Question B11: “I was standing in several places at the same time”; and Question 12: “I perceived the virtual interior space and myself from the outside”) (Slater, 2009); sensation of *movement* (Question B3: “I was moving along the interior space”); sensation of *depth* (Question B4: “Certain areas within the interior space were located further away from me”; Question B5: “The side walls were located closer to me than other parts of the virtual interior space”; Question B7: “The ceiling and ground were located very close to me”; and Question B8: “Every element of the interior space was equally far from me” (see **Figure 3A**, left); as well as sensation of *touch* [Question B6: “Some elements enclosing the virtual interior space were touching my body” (Pasqualini et al., 2013)] (**Table 1B**). All questions were rated on a scale from 1 to 10, where 1 indicated strong disagreement and 10 strong agreement.

#### Procedure

Participants were placed at the center of the tracking arena facing the screen (**Figure 3A**) and they were asked to fixate in the direction of the avatar. The experimenter was standing behind them to conceal the stroking procedure from their vision. Using a trackable stick, they were stroked on the back for 2 min consecutively while on the screen they saw either the back of the avatar or the front wall stroked in a synchronous or asynchronous way. The distance between the displayed virtual walls corresponded to a large or a narrow interior space. We used a two-by-two factorial design with stimulation (Synchronous and Asynchronous) and interior (Large and Narrow) as within-factors. Thus, each participant was exposed to four experimental conditions, administered in counterbalanced order. White noise masking stroking-related noise was presented to the participants over headphones to isolate them from the physical environment. Before exposing each participant to the four experimental conditions, we explained the procedure carefully. After each block of visuotactile stimulation, we administered the questionnaires. The four conditions were randomized across participants. The order of questions in the two questionnaires at the end of each condition was also presented in a random order among participants. Participants took a short break before each condition.

#### Data Analysis

To analyze the questionnaire responses with a factorial design, we firstly standardized participants’ ratings using ipsatization procedure (Cattell, 1944; Broughton and Wasel, 1990; Slater et al., 2008; Tsakiris, 2010). Specifically, the mean score of the participants’ responses to all questions and conditions was subtracted to each question score and then divided by the standard deviation of participants’ responses to all questions and conditions. This approach has been used in several other papers whereby questionnaire ratings were analyzed in a multi-factorial design (Romano et al., 2014; Ronchi et al., 2015; Blefari et al., 2017). Ipsatized scores were then analyzed utilizing two-way repeated measures ANOVA, with *stimulation* (Synchronous and Asynchronous) and *interior* (Large and Narrow) as within-factors.

### Experiment 2

#### Participants

A sample of 9 healthy, right-handed participants different from those recruited in Experiment 1 was considered for Experiment 2 (mean age 21.4 ± 0.9 years; 4 female). The effect size for Experiment 2 was calculated on the basis of the results of Experiment 1 for the same question included in the two experiments, that is question A1 (referred touch question). An effect size of N > 4 and N > 5 was estimated based on the difference between the synchronous and asynchronous conditions from Experiment 1 in the large and narrow condition respectively (with a power respectively of 0.98 and 0.96). We doubled the required sample size in order assure enough power.

#### Materials

The only difference between the setup in Experiment 2 and Experiment 1 was that the visual stroking was applied to the virtual front wall instead of the avatar (**Figure 3C**). All the other study parameters were equivalent.

In Experiment 2 we used a shorter eight items questionnaire, combining six questions of the bodily self-consciousness questionnaire and two questions of the architecture questionnaire of Experiment 1. We extracted only those questions from the previous study that could be adapted to the virtual interiors without an avatar (**Table 1C**). The questions that could not be adapted to a scene without an avatar were discarded, along with redundancies in the architecture questionnaire. Thus, only questions focusing on somesthetic experience were kept. In particular, we considered questions measuring *tactile sensation* (Question A1: “I could locate the touch of the stick in the location where I saw the virtual wall being touched”; Question A2: “The touch I felt was caused by the stick touching the virtual wall”; Question A6: “The touch I felt came from somewhere between my own body and the virtual front wall”); *self-identification* (Question A3: “The virtual interior space was part of me”); as well as *self-location* (Question A4: “My physical body was drifting toward the virtual front wall”); and the control question (Question A8: “I was in two places at the same time”) (**Table 1A**). Most importantly, given that in the setting of Experiment 2 touch was applied on an empty space at a distance from the participants, it seemed possible that participants would agree to a statement such as Question A6, and therefore it could not be considered a control question any more. Thus, we tested whether this effect occurred more commonly in the synchronous than the asynchronous condition. The remaining architecture questions were related to *touch* (Question B6: “Some elements enclosing the virtual interior space were touching my body”), and, as a further control question, *presence* (Question B9: “I was standing in an open space”) (**Table 1B**). As in Experiment 1, all items were rated on a scale from 1 to 10, where 1 indicated strong disagreement and 10 strong agreement.

#### Procedure

We adopted the same experimental procedures as in Experiment 1.

#### Data Analysis

We performed the same data analysis as in Experiment 1.

## Results

### Experiment 1

**FIGURE 4:**
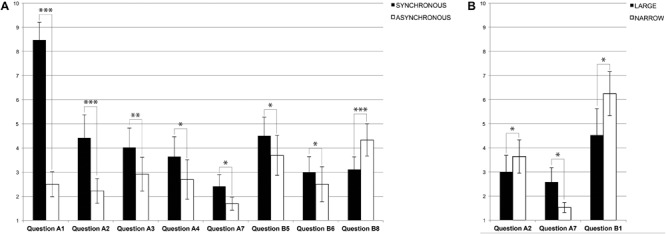
Experiment 1 – Questionnaires. **(A)** Significant main *stimulation* effects in Experiment 1 from ANOVAs in the self-consciousness A and architecture B questionnaires (^∗^*p* < 0.05; ^∗∗^*p* < 0.01; ^∗∗∗^*p* < 0.005). The plots are based on the average response calculated with non-normalized data; error bars represent standard errors. **(B)** Significant main *interior* effects in Experiment 1 from ANOVAs in the self-consciousness and architecture questionnaires (^∗^*p* < 0.05). The plots are based on the average response calculated with non-normalized data; error bars represent standard errors.

In the bodily self-consciousness questionnaire (**Table 1A**) we found a main effect of *stimulation* (with higher ratings in the synchronous as compared to asynchronous stimulation) for *visuotactile congruence* [Question A1: “I located the touch of the stick where I saw the virtual body being touched”; *F*(1,17) = 83.77, *p* = 0.000]; *referred touch* [Question A2: “The touch I felt was caused by the stick touching the virtual body”; *F*(1,17) = 15.79, *p* = 0.000]; and *self-identification* [Question A3: “The virtual body was my body”; *F*(1,17) = 10.26, *p* = 0.005]. This analysis also revealed illusory changes in the location of the own physical body with respect to the avatar (larger in synchronous versus asynchronous stimulation), characterized by a significant forward drift in self-location [Question A4: “My physical body was drifting toward the front (toward the virtual body)”; *F*(1,17) = 7.98, *p* = 0.011]; and, the illusory backward drift of the avatar [Question A7: “The virtual body was drifting backward (toward my own body)”; *F*(1,17) = 5.79, *p* = 0.027] (**Figure 4A**). The architectonic embodiment questionnaire (**Table 1B**) revealed a main effect of *stimulation*, with stronger ratings for *containment* or wall retraction during synchronous as compared to asynchronous stimulation [Question B5: “The sidewalls were located closer to me than other parts of the virtual interior space”; *F*(1,17) = 6.06, *p* = 0.024]. Ratings were lower for illusory *touch* sensation, but also, in this case, they were significantly higher in the synchronous versus asynchronous condition [Question B6: “Some elements enclosing the virtual interior space were touching my body”; *F*(1,17) = 6.22, *p* = 0.023]. Conversely, the feeling that all surfaces were at the same distance from the physical body was stronger for asynchronous stimulation (Question B8: “Every element of the interior space was equally far from me”; *p* = 0.000) (**Figure 4A**).

A main effect of the *interior* was observed in the bodily self-consciousness questionnaire for *referred touch* [Question A2; *F*(1,17) = 6.39, *p* = 0.021], with a higher score for the narrow interior. We also found that drift or illusory *self-location* [Question A7; *F*(1,17) = 6.49, *p* = 0.020] was rated higher for the large interior space (**Figure 4B**). In the architectonic embodiment questionnaire, a significant main effect of the *interior* was found to the question addressing *place* perception, with higher response in the narrow condition [Question B1: “I was standing inside a corridor”; *F*(1,17) = 5.71, *p* = 0.028] (**Figure 4B**). None of the other questionnaire items showed significant main effects nor interactions.

Viewing an avatar being touched within a virtual interior, while standing within a Virtual Reality arena and receiving homologous tactile stimulation on one’s body, induced specific changes in both, bodily self-consciousness, and, the experience of the environment. Participants experienced changes in their subjective sensation of self-location. The dimensions of the virtual interior altered the perceived continuity between physical and virtual space, abolishing the view of the virtual extension for asynchronous stroking. On the other hand, the full-body illusion also influenced the way how close people were “feeling” to the virtual interior and its enclosing walls. In Experiment 2, we inquired whether these effects depended on the presence of an avatar with which participants identified inside a given interior, or, rather, whether they were not simply due to the temporal pattern of synchronous, as contrasted to asynchronous visuotactile stimulation.

### Experiment 2

**FIGURE 5:**
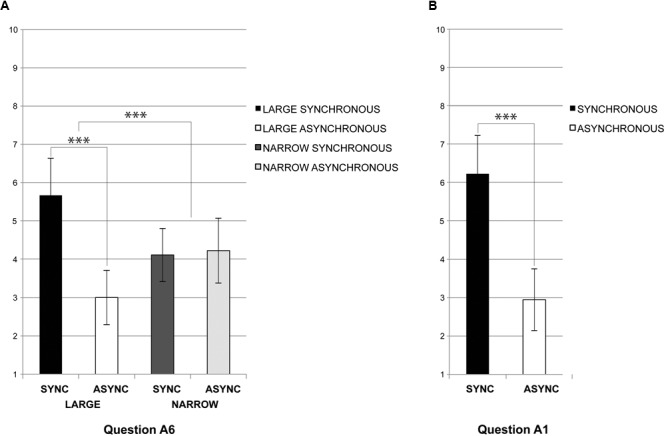
Experiment 2 – Questionnaires. **(A)** Significant two-way interaction effects between *stimulation* and *interior* in Experiment 2 from ANOVAs in self-consciousness Question A6: “The touch I felt came from somewhere between my own body and the virtual enclosure.” (^∗^*p* < 0.05; ^∗∗^*p* < 0.01; ^∗∗∗^*p* < 0.005). The plots are based on the average response calculated with non-normalized data; error bars represent standard errors. **(B)** Significant main effects of *stimulation* in Experiment 2 from ANOVAs in self-consciousness Question A1, *visuotactile congruency effect*: “I could locate the touch of the stick in the location where I saw the virtual wall being touched.” (^∗^*p* < 0.05; ^∗∗^*p* < 0.01; ^∗∗∗^*p* < 0.005). The plots are based on the average response calculated with non-normalized data; error bars represent standard errors.

When the same experiment was repeated without avatar, but with visuotactile stroking applied to the front wall of the virtual interior, we found a two-way interaction between *stimulation* by *interior* for *referred touch* sensation [Question A6: “The touch I felt came from somewhere between my own body and the virtual enclosure”; *F*(1,8) = 15.72, *p* = 0.003], showing a synchronous-asynchronous difference in the large, but not in the narrow room condition. In addition, a main effect of *stimulation* with higher scores in the synchronous condition was found for *visuotactile congruence* [Question A1: “I could locate the touch of the stick in the location where I saw the virtual wall being touched”; *F*(1,8) = 16.61, *p* = 0.004] (**Figures 5A,B**). No other question was significant.

**FIGURE 6:**
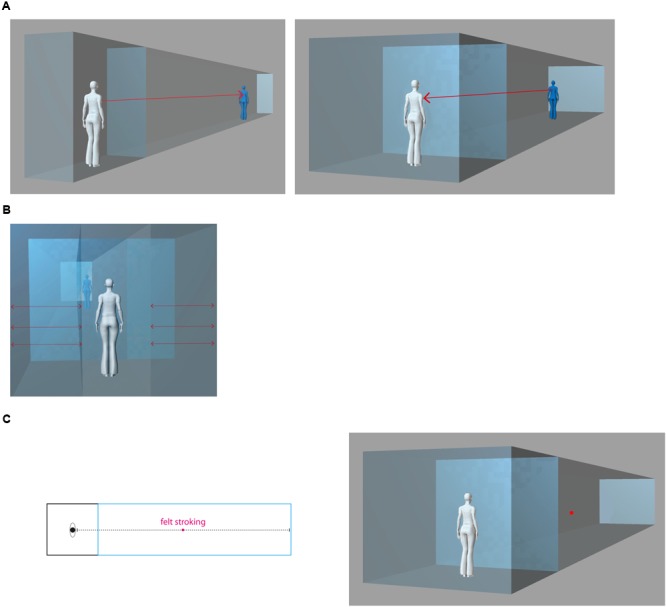
Architectonic self-consciousness. The subjective perception of depth was significantly modulated by a referred somatosensory sensation toward the virtual interior evoked by the avatar. The presence of the avatar altered the perception of both, body and space in the augmented interiors. **(A)** Own body drifting toward the avatar depended in Experiment 1 only on *stimulation* with higher responses for synchrony (Question 4); the backward drift of the avatar was induced by *stimulation* and *interior* with higher responses in large and synchrony (Question A7). **(B)** The Full-Body-Illusion in Experiment 1 induced a sensation of contraction (Question B5) and touch (Question B6) through the sidewalls evoked by *stimulation.* **(C)** The touch was felt halfway between the physical body and the virtual front wall (Question A6) depending on an interaction between *stimulation* and *interior* for large and synchrony in Experiment 2.

Thus, presenting visuotactile stimulation in the absence of the avatar abolished most effects related to bodily self-consciousness and the illusions, and only the multisensory effects related to the spatial location of the visuotactile stimulus was preserved, as well as a partial interaction effect of referred touch. In Experiment 1, Question 6 was considered a control question. Since in the absence of the avatar it could be assumed that participants would locate the touch somewhere between themselves and the front wall, if, and only if they experienced a virtually augmented depth. In the large interior, participants reported intense sensations of the stroking instrument being suspended in-between the virtual front wall and their physical body during the full-body illusion (**Figures 5A, 6C**). This interaction of factors suggests that the interior dimension is a necessary condition to define a substantial volumetric sensation of immersion ranging from somewhere between “my own body and the virtual enclosure” through visuotactile synchrony. Participants perceived the stroking instrument as if floating within a static void. At the same time, the absence of the avatar abolished feelings of *drift, touch*, and *containment*, like those reported in Experiment 1. We found no further effects of *referred touch* with the front wall or *self-identification* with the interior space.

## Discussion

The present paper explores perceptual processes of embodiment in augmented interior spaces based on evidence from bodily self-consciousness studies (Lenggenhager et al., 2007; Longo et al., 2008; Aspell et al., 2009). Several aspects of bodily self-consciousness can be altered through multisensory manipulations inducing changes of self-identification and self-location. With our experiments, we investigated the mutual relationship between architectonic space and body space through changes in bodily self-consciousness. We studied whether the effect of manipulation of multisensory bodily cues (visuotactile stroking) on bodily self-consciousness varied in function of the characteristics of the architectonic space (narrow vs. large interior). In turn, we assessed how such manipulation altered the ways participants perceived the architectonic space, and themselves inside those interiors. Although embodiment is recognized as an architectonic phenomenon, and, empirical evidence suggests that the influence of architectonic space on bodily self-consciousness can be quantified (Pasqualini et al., 2013), experimental studies about perceptual, cognitive, affective and motor mechanisms on human participants remain sparse.

Previous works have shown the relevance of Virtual Reality to test environmental effects on bodily self-consciousness (Slater, 2009). Different from classical representations (e.g., linear perspective, see **Box 1**) in which egocentric, first-person perspective could not be dissociated from the physical point of view without losing the unity of perception, visuotactile illusions generate an egocentric view that is not bound to the physical body anymore but to the avatar. Experiment 2 was specifically designed to test whether the somesthetic changes in bodily self-consciousness and interior perception depended on the presence of the avatar. To this aim, we presented visuotactile stroking on the front wall of the same interiors, thus without the virtual body (**Figure 3C**).

Findings show a partial modulation of the subjective experience of space. Evaluation of the main questionnaire items resulted in scores increase of self-identification with the avatar, independent of the room in which it was presented. During the full-body illusion, referred touch was rated higher for the narrow space. We also found several visuospatial and somesthetic illusions in the large and narrow space, such as drifting of the own body in space, containment, and touch. The qualitative aspects of these depth sensations were likely modulated by the presence of the avatar. However, when the latter was absent, participants perceived a spatial continuity in the large space.

### Experiment 1

As expected from previous work on rubber-hand-and full-body illusions (Botvinick and Cohen, 1998; Ehrsson, 2007; Lenggenhager et al., 2007, 2009; Tsakiris et al., 2007; Petkova and Ehrsson, 2008; Aspell et al., 2009; Slater et al., 2010; Tsakiris, 2010), the present findings show that participants self-identified with the avatar in the virtual interior depending on the synchrony of visuotactile stimulation. This outcome was associated with stronger feelings of touch and depth for the augmented interior. We also found two subjective responses (illusory drift toward the avatar and of the latter backward) compatible with a decrease of the virtual boundary between participant and avatar. Previous full-body illusion studies reported these effects partially (on illusory drift and self-location see Lenggenhager et al., 2009; Serino et al., 2013; Noel et al., 2015; Salomon et al., 2017). We argue that these visuospatial alterations were enforced by the perspective cues and are mainly related to an extension of the boundaries of peripersonal space toward the avatar and the virtual interior. That is, the tactile sensation on one’s own body synchronized with the visual stimulation of the avatar’s body extended the receptive field for which the visuotactile cues were integrated toward the whole interior (see Noel et al., 2015; for review see Blanke et al., 2015). These effects elicit a drift sensation also involving other multisensory cues, such as vestibular inputs with visuospatial effects (Graziano et al., 1997; Ionta et al., 2011; Pfeiffer et al., 2013, 2014a).

The present setup differs from the majority of the full-body illusion studies, where the own filmed body was sometimes introduced only partially on a Head-Mounted-Display as an avatar bare of any environmental information (see e.g., Lenggenhager et al., 2007). Here, as illustrated in **Figure 3**, we presented a computer generated full-body avatar, embedded into two-dimensional virtual interiors projected in perspective on the screen of a Virtual Reality arena. In this way, the perspective angle of a recording device (video camera) did not limit our participants’ field of view, nor did a Head-Mounted-Display conceal the vision of the physical body or flatten digital space (Mohler et al., 2010). Both, the physical and the virtual body were perceived together within a continuous interior through visuotactile stroking. The present changes in bodily self-consciousness suggest that the full sight of the own physical and the virtual body with spatial cues enforces changes in subjective self-location.

Furthermore, the present results show that the size of the interior where the avatar was presented also modulated the subjective experience independent from the full-body illusion. We found that exposure to the narrow interior enforced referred touch sensations from the participants’ physical body toward the avatar’s location, whereas in the large interior an increased illusory backward drift of the avatar was observed (**Figures 4B, 6A**). These effects appear to be complementary and support the hypothesis of an influence of the spatial cues on self-location, in the sense that the closer side walls promoted tactile sensations toward the direction where participants saw the touch, as if to activate a potential visuomotor affordance (Gibson, 1979/1986; Oztop and Arbib, 2002). In the large condition, we did not find such effect, since the visual stimulus (i.e., the avatar) was projected backward, toward the volume of somatosensory stimulation. In this context, it seems that visuotactile mechanisms respond to an expected touch with a behavior of estimation, relative to a pattern of proximity (see also Noel et al., 2018).

Sensations of drift with illusory touch during the full-body illusion support the experience of a bidirectional shift of self-location between the physical and the virtual interior. Such sensations were differently impacted by the size of the interiors, pointing to ambivalent depth sensations at the boundary between extrapersonal and peripersonal space. Specialized brain regions map different sectors of space, by integrating various sources of information and body part movements through a dissociation between extra- and peripersonal space (Rizzolatti et al., 1983; Bisiach et al., 1986; Halligan and Marshall, 1991). Peripersonal space is mapped through the multisensory integration of bodily inputs related to external objects including tactile, proprioceptive and vestibular signals with visual and auditory cues. In contrast, ‘distal’ senses, such as vision and audition, more actively contribute to the mapping of extrapersonal space (Rizzolatti et al., 1981a,b; Grüsser and Landis, 1991). By manipulating spatiotemporal coherence (visuotactile synchrony) between somatosensory and visual cues in space, it was possible to affect the subjective perception of oneself in space. More specifically, the multisensory conflict between touch in the peripersonal space and the synchronous visual cues from the extrapersonal space was presented in a spatial context that affected the way, in which the conflict was resolved. The narrow interior favored the somesthetic experience of the virtual side walls (see also the corridor effect for narrow in question B1); while the large interior that of the interior volume through a more global and visual depth sensation. The architecture questionnaire shows that in the synchronous condition participants felt as if some elements of the interior were touching their physical body (**Figures 4A, 6B**, Question B6) and as if the sidewalls of the interior were approaching them (**Figures 4A, 6B**, Question B5). Conversely, in the asynchronous condition, they perceived the elements of the interior space as equally distant from their body (**Figure 4A**, Question B8), matching their physical location to the physical arena and not to the augmented continuum.

These responses suggest that in the asynchronous condition participants perceived themselves in the physical environment more than in the virtual one (as shown in **Figure 3A**). Based on our previous findings using a full-body illusion with a Head-Mounted-Display, we expected stronger effects of touch illusion only in the narrow condition, where an avatar was necessary to convey a sensation of depth (Pasqualini et al., 2013). Instead, in the present experiment, the main effects of the temporal pattern of stimulation prevailed over those of the interior (**Figures 4A,B**). We believe that the view of the physical body in first person in the augmented physical interior induced such outcomes. Overall, the main effects of multisensory stimulation dominated the main effects of context. Ownership feelings for the avatar mediated a situated sensation of place between the physical body and the virtual interior, as if the presence of the avatar, and its embodiment through synchronous multisensory stimulation, acted as a trigger for affordances inside the virtual interior, activating potential sensorimotor interactions.

### Experiment 2

The comparison between the first and the second experiment reveals that the vast majority of the effects depended on the virtual body. Particularly, in Experiment 1 multisensory processes were enhanced by the walls in the proximity of the avatar inducing a subjective sensation of shift of one’s bodily space toward the virtual space. In the absence of the avatar, most of these effects disappeared, and synchrony had a much weaker effect on visuotactile congruence (**Figure 5B**, Question A1). Participants perceived the continuity of space between physical and virtual space only in the large interior and during visuotactile synchrony. In Experiment 2, we found no evidence for ownership or self-identification with any of the elements shown in the virtual space, comparable to one of the control experiments performed by Lenggenhager et al. (2007), where self-identification was not reproduced when the avatar was replaced by a human-sized box. This result also concurs with previous studies on the Rubber-Hand-Illusion (Tsakiris and Haggard, 2005; Hohwy and Paton, 2010; Ma and Hommel, 2015a,b); but see Armel and Ramachandran (2003).

There is a mutual relationship between the perception of magnitude in different sensory modalities and body size representations. It has been shown that the manipulation of the perceived size of body parts by means of multisensory processing (visual, tactile, proprioceptive, vestibular) alters tactile and visual perception in a bottom-up way (Taylor-Clarke et al., 2004; de Vignemont et al., 2005; Serino and Haggard, 2010; Linkenauger et al., 2011; Van der Hoort et al., 2011; Banakou et al., 2013). Depending on such constantly updated three-dimensional volumetric experience of one’s physical body, we argue that here the visuotactile effects activated a more visual nuance of spatial experience. In the previous experiment, the ambiguity between extra-and peripersonal space, shown by shifts of self-location between physical and virtual interior, had a somatosensory component through the identification with the avatar. Here, the effects of visuotactile stimulation with the front wall were not sufficient to induce the previously reported changes of multisensory perspective in both, large and narrow space. In contrast, synchronous visuotactile stimulation induced a displacement of tactile sensation of depth toward the suspended stroking instrument inside the virtual interior volume.

Interestingly, this transfer of body sensation toward the virtual interior during synchronous stimulation was significant only for the large space, not the narrow one. Why? These findings complement the results of our former study (Pasqualini et al., 2013), in which self-identification with an avatar increased depth perception only when performed in the narrow interiors during the full-body illusion, a score that was equivalent to both conditions of synchrony in the large interior. We thus argue for a multisensory embodiment of the sidewalls through an illusory lateral touch on the arms and shoulders of the avatar in the narrow condition. In conclusion, we found two separate modalities of transposition of body sensation toward the virtual interior, a somatosensory activation concerning the whole volume of the body in the large space, and a sensorimotor affordance induced by the narrow space, that was only perceived as an interior in the presence of the avatar. This perceptual modulation could explain why in the first experiment visuotactile synchrony evoked illusory touch also for the narrow condition. On the other hand, the sensation of continuity in the large space appears to build rather on volumetric aspects of space linked to visual, multisensory and somesthetic processes, as described previously by Pfeiffer et al. (2014b).

## Conclusion

William James proposed that the “original sensation of space” – described as the genuine “sensation of volume” – builds the foundation of self-consciousness based on the unified and continuous presence of the bodily self as the background of human action (James, 1890/1950). We suggest that much in the same way architectonic interiors may be processed as a second volume or *Gewand*, supporting, incorporating, and locating our bodily space during daily interactions and perceptions, as something which is always there – around us. In Schmarsow’s architecture, space is revealed by a shifting point of view *within* the architectonic volume – an effect supposed to emphasize a global sensation of space that is perceived through the whole body, and which determines a moment of presence situated *in space and time*. Thus, for Schmarsow the immersive experience of space, mapping the architectonic environment *from within* and mediating somesthetic mechanisms, favors the perception of the immediately surrounding interior as part of one’s peripersonal space, whereas distal cues favor the processing of the visual and less interactive extrapersonal space. In our experiments, we found evidence for a self-conscious modulation of interior space perception based on the presence of an avatar, including a weaker and partial effect for the void space. It seems that, as predicted by Schmarsow’s theory, the integration of multisensory and somesthetic cues between peripersonal and extrapersonal space is relevant for the experience of architectonic space. Wölfflin (**Figure 2B** and **Box 2**), described a related but distinct mechanism. Wölfflin locates his observer *in front* of the architectonic structure in an embodied first-person view. From our data, it appears that the avatar enabled a shift toward virtual space, related to sensorimotor mechanisms when the walls were presented closer to the body. The human body seems to point beyond the mere functionality of the metric size cue introduced as a measure of scale in architectural drawings and paintings but might represent an element of embodiment that enables to extend the experience of oneself into the augmented, architectonic or pictorial interior.

Recent publications raise compelling arguments about mutual concepts of embodiment in architecture and neuroscience (Eberhard, 2009; Mallgrave, 2013; Pasqualini and Blanke, 2014). A commonplace to the theories of embodiment has been related to the search for the origin of meaning in architectonic space linked to a human need for beauty and collective social behavior or emotions (Mallgrave, 2015). Neuropsychological studies on right brain-damaged patients suffering from visual agnosia for places, or topographic disorientation, show that neurobiological mechanisms have a great influence on spatial sensations and mood (Landis et al., 1986; Habib and Sirigu, 1987; Grüsser and Landis, 1991; Aguirre and D’Esposito, 1999). Moreover, it was found that hippocampal place cells in humans respond differently, whether exposed to visual stimuli of landmarks, objects, room interiors, urban interiors or landscapes, and, that these stimuli influence environmental behavior and learning (Epstein and Kanwisher, 1998; Epstein et al., 1999). The results of the present experiments show that multisensory aspects of space are both, linked to embodiment and the sensation of volume (Lopez et al., 2008; Tsakiris et al., 2008). Depth feelings emerge through different modulations, on the one hand through stimuli presented in peripersonal space of a physical or virtual body, or, somesthetic processes linked to the perception of a volume and continuity. This makes sense, as the unity of bodily experience against a set of evolving background conditions is a fundamental requirement of human interaction with the environment. The way in which the environment offers more variation, affects the qualitative impact of space on humans.

## Author Contributions

IP contributed as first author to the writing and conception of both studies, participants’ data collection and analysis, as well as all the figures. MB contributed to the statistical analysis of both studies and provided intense proof-reading of the manuscript. TT contributed in both studies to the conception as well as to collecting the participants’ data. OB and AS contributed in equal parts to the conception of both studies, data analysis, and writing.

## Conflict of Interest Statement

The authors declare that the research was conducted in the absence of any commercial or financial relationships that could be construed as a potential conflict of interest.
